# A Combination of Radiotherapy, Hyperthermia, and Immunotherapy Inhibits Pancreatic Tumor Growth and Prolongs the Survival of Mice

**DOI:** 10.3390/cancers12041015

**Published:** 2020-04-21

**Authors:** Javed Mahmood, Allen A. Alexander, Santanu Samanta, Shriya Kamlapurkar, Prerna Singh, Ali Saeed, France Carrier, Xuefang Cao, Hem D Shukla, Zeljko Vujaskovic

**Affiliations:** 1 Division of Translational Radiation Sciences (DTRS), Department of Radiation Oncology, University of Maryland School of Medicine, Baltimore, MD 21201, USA; allenabey.alexander@gmail.com (A.A.A.); santanu.samanta@umm.edu (S.S.); shriya.kamlapurkar@gmail.com (S.K.); prerna.singh@umaryland.edu (P.S.); alisaeed@umm.edu (A.S.); FCarrier@som.umaryland.edu (F.C.); ZVujaskovic@som.umaryland.edu (Z.V.); 2 Department of Microbiology and Immunology, University of Maryland School of Medicine, Baltimore, MD 21201, USA; XuefangCao@som.umaryland.edu; 3 Department of Neurology and Neurosurgery, Johns Hopkins University, School of Medicine, Baltimore, MD 21205, USA; hshukla2@jhmi.edu

**Keywords:** pancreatic cancer, immunotherapy, hyperthermia, radiation therapy, combination treatment, Anti-OX40

## Abstract

Background: Pancreatic cancer (PC) is the fourth-most-deadly cancer in the United States with a 5-year survival rate of only 8%. Unfortunately, only 10–20% of PC patients are candidates for surgery, with the vast majority of patients with locally-advanced disease undergoing chemotherapy and/or radiation therapy (RT). Current treatments are clearly inadequate and novel strategies are crucially required. We investigated a novel tripartite treatment (combination of tumor targeted hyperthermia (HT), radiation therapy (RT), and immunotherapy (IT)) to alter immunosuppressive PC-tumor microenvironment (TME). (2). Methods: In a syngeneic PC murine tumor model, HT was delivered before tumor-targeted RT, by a small animal radiation research platform (SARRP) followed by intraperitoneal injections of cytotoxic T-cell agonist antibody against OX40 (also known as CD134 or Tumor necrosis factor receptor superfamily member 4; TNFRSF4) that can promote T-effector cell activation and inhibit T-regulatory (T-reg) function. (3). Results: Tripartite treatment demonstrated significant inhibition of tumor growth (*p* < 0.01) up to 45 days post-treatment with an increased survival rate compared to any monotherapy. Flow cytometric analysis showed a significant increase (*p* < 0.01) in cytotoxic CD8 and CD4+ T-cells in the TME of the tripartite treatment groups. There was no tripartite-treatment-related toxicity observed in mice. (4). Conclusions: Tripartite treatment could be a novel therapeutic option for PC patients.

## 1. Introduction

Pancreatic cancer (PC) is the fourth most lethal cancer in the United States. In 2020, there will be an estimated diagnosis of 57,600 new cases, and 47,050 deaths [1]. Owing to lack of sensitive biomarkers for early diagnosis, the disease is normally diagnosed in the advanced stages, and less than 20% of diagnosed patients have the option to undergo surgery. Further, the treatment options for the approximately 40% of locally-advanced pancreatic cancer (LAPC) patients that are not qualified for resectable surgery are being continuously developed. The current treatment paradigms for pancreatic cancer involving surgery, chemotherapy, and/or radiation therapy (RT) have certainly shown survival improvements, but only for a margin of patients (i.e., resectable, margin-negative patients), and median survival has remained low [2,3,4,5,6]. Therefore, novel therapeutic advances or improved treatment modalities are desperately needed. In the present study, we have examined whether RT could be augmented by combining immunotherapy and hyperthermia [7,8,9,10,11,12] to treat locally-advanced pancreatic cancer (LAPC).

**Figure 1:**
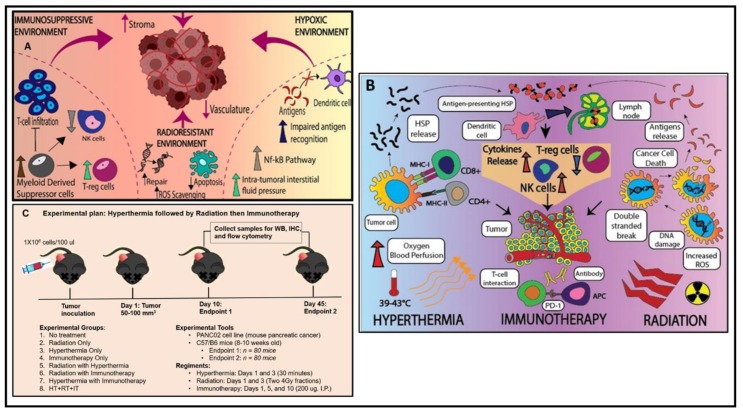
(**A**) Schematic representation of immunosuppressive tumor microenvironment (TME) of pancreatic cancer (PC). This immunosuppressive TME is characterized by increased myeloid-derived suppressor cells, increased T-regulatory (T-reg) cells and decreased natural killer cells (NK). PC-TME is highly hypoxic, less vascular, rich in stroma, resulting in radioresistant microenvironment. PC-TME also has increased tumor interstitial fluid pressure (IFP), activated NF-kappa B pathways, and impaired antigen recognition which causes decreased activated dendritic cells. (**B**) Proposed mechanism of tripartite treatment in pancreatic cancer which alters the PC-TME. Radiation causes DNA-double-strand break either by direct damage but also through free radicals generated in cells—mostly the -OH radical from water. Increased reactive oxygen species (ROS) causes increased tumor cell apoptosis that increases natural killer cell (NK) cells which could kill tumor cells in the TME. Hyperthermia increases perfusion and blood flow in the TME. Hyperthermia also increases tumor cells’ expression of heat-shock proteins (HSPs) and antigens and they then become antigen-presenting cells that trigger dendritic cells to travel to the lymph nodes which causes T-cell activation. OX40 agonist binds with OX40 ligand on the CD8 T-cells and activates them and that releases increased granzymes and perforins that kill tumor cells in the TME. (**C**) Anti-OX40 immunotherapy (IT), radiation therapy (RT), and hyperthermia (HT) tripartite treatment experimental plan in pancreatic cancer pre-clinical study.

Among many challenges posed by PC that hinder treatment, a key obstacle is a highly stromal tumor microenvironment (TME) (Figure 1A) [13]. The dense stroma prevents penetration of chemotherapeutic drugs and anti-tumor immune cells. In addition to presenting a physical barrier, the resident fibroblasts and inflammatory cells within the stroma create an immunosuppressive TME, hindering the ability of the immune system to recognize and eliminate tumor cells [10,13,14,15,16]. Efforts to reverse this immunosuppressive TME may be key for effective treatment (Figure 1B).

RT, in addition to directly inducing cytotoxicity through DNA damage, can also powerfully alter and reverse the immunosuppressive TME [10]. RT is known to upregulate antigen presentation, induce neo-antigens, increase cytotoxic T-cell infiltration, and downregulate T-reg cells [8,9,10]. We have examined the ability of tumor-targeted hyperthermia and immunotherapy to augment RT-induced immune stimulation. Hyperthermia (HT), in addition to enhancing RT effects through reoxygenation and by inhibiting DNA repair, can also target the immunosuppressive TME. HT is known to induce vasodilation, which allows for infiltration of immune cells. Additionally, hyperthermia increases immunogenic surface receptors such as NKG2D and ligand-like MHC-I and it promotes expression of heat-shock proteins (HSP) for enhanced antigen presentation [17,18,19]. Though RT and hyperthermia exert pro-immune effects on the TME, those responses can likely be further directly enhanced with immune stimulation using immunotherapy agents. Immunotherapy, in the form of check-point inhibitors targeting CTLA4 and PD1, has shown remarkable responses in select malignancies, but has not shown clinical efficacy for pancreatic cancer [20,21,22,23,24]. We therefore examined a different class of immunotherapy, focusing on OX40 activation via agonistic antibody [25,26]. OX40 is not a checkpoint regulator, but rather directly stimulates T-cell activation by functioning as a co-stimulatory signal that leads to T-cell activation, clonal expansion, memory function, and cytokine production. Agonist OX40 antibody is in early-phase clinical trials and has shown efficacy in preclinical models [26].

As described, RT, hyperthermia, and immunotherapy can function via different mechanisms (Figure 1B) that ultimately converge to help reduce the immunosuppressive nature of the TME associated with pancreatic cancer [10] and make it more immune responsive. In the present study we tested the ability of combination therapy consisting of RT, hyperthermia, and OX40 immunotherapy (called tripartite treatment) to inhibit pancreatic tumor growth in a syngeneic mouse model and examined the effects within the TME.

## 2. Results

In the present study, tripartite treatment was designed to maximize the synergistic effects, and concomitantly minimize the toxicities associated with an individual treatment.

### 2.1. Tripartite Treatment Augments the Anti-tumor Response

**Figure 2:**
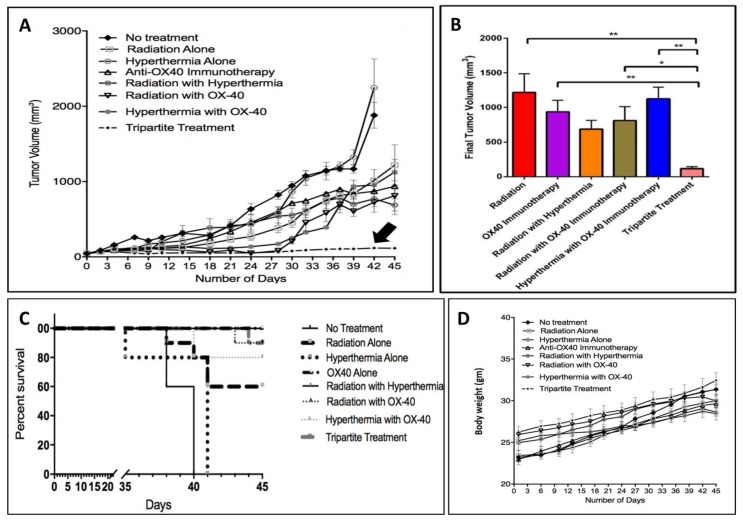
Longitudinal growth of PC tumors in a syngeneic mouse model. (**A**) Longitudinal tumor growth in PC-tumor-bearing mice. Arrow indicates the tripartite treatment. (**B**). Tumor growth difference of PC-tumor-bearing mice at 45 days. Histogram demonstrating the difference in tumor volume between the group of animals treated with radiation versus the combination of the treatments (*n* = 10), ‘*n*’ demonstrates number of animals per group (mean ± SEM). * *p* < 0.05, ** *p* < 0.01. (**C**) Kaplan–Meier survival plot for PC-tumor-bearing animal cohorts treated with individual treatments and in different combinations of anti-OX40 IT, RT, and HT. Tripartite treatment showed the *p*-value of *p* < 0.01 compared to un-treated control. (**D**) The body weight plot of PC- tumor-bearing mice cohorts over 45 days of study.

Using a syngeneic mouse-tumor model with subcutaneously implanted panc02 cells, we tested the efficacy of tripartite treatment of combined hyperthermia (HT), radiation therapy (RT), and OX40 immunotherapy (IT). We compared the tripartite treatment with each monotherapy individually (i.e., HT-alone, RT-alone, and IT-alone). In addition, tripartite treatment was also compared with combinations of two therapies (bipartite) (i.e., RT + HT, RT + IT, and HT + IT), resulting in a total of seven treatment groups. These treatment groups were directly compared against control (i.e., no treatment). Figure 2A shows tumor volumes for the various treatment groups over the span of 45 days, which was the end of the study. The tripartite treatment group demonstrated a significant tumor volume reduction (Figure S2) and remarkable anti-tumor response compared to control, and all other treatment groups (Figure 2A). Further, animals treated with RT alone or IT alone (monotherapy), also exhibited significant tumor regression (*p* < 0.05) compared to control (no treatment animals). However, RT and IT as an individual therapeutic approach did not show any superior therapeutic effect of tumor regression potential compared to each other or tripartite treatment. We also evaluated effectiveness of bipartite treatments (i.e., RT + HT, RT + IT, and HT + IT) to inhibit tumor progression. The data in Figure 2A–C have shown that RT + HT, RT + IT, and HT + IT also exhibited significant tumor control compared to control (no treatment group) (*p* < 0.01, *p* < 0.05, *p* < 0.05, respectively). Conversely, HT-alone showed a trend of increased tumor progression but was not statistically significant compared to control animals. Interestingly, the most striking tumor growth inhibition was observed in the tripartite treatment, with greater inhibition than the monotherapy, bipartite, or control groups (Figure 2A–C). Figure 2A,B show the actual tumor size following euthanasia at 45 days post treatment initiation. All treatment group showed significant tumor weight reduction (*p* < 0.05) at 45 days compared to non-treated control animals (Figure S3).

### 2.2. Tripartite Treatment Increases Animal Survival with No Treatment-Related Toxicity

We examined the effect of tripartite treatment on animal survival. As depicted in Figure 2B, the tripartite treatment exhibited a 90% survival of animals over the 45-day course of the experiment. In comparison, amongst the animals receiving no treatment and HT-alone, 100% mortality was observed by days 40 and 42 respectively (Figure 2B). All other treatment groups demonstrated mortality except for the IT-alone group (100% survival). Furthermore, animals receiving a combination of HT + IT showed 80% survival at the study endpoint. Interestingly, the RT-alone group showed 60% survival, whereas RT + IT showed 90% overall survival. Overall, IT alone or in combination with HT or RT or as part of tripartite treatment demonstrated survival benefit to animals with PC. Importantly, tripartite treatment was well tolerated with no body weight loss (Figure 2C) observed in those mice. Furthermore, none of the mice in any treatment group demonstrated more than 20% of body weight loss throughout the course of the study (Figure 2C).

### 2.3. Tripartite Therapy Potentiates Tumor-Targeting T-Cell Infiltration and Activation in the Tumor Microenvironment

**Figure 3:**
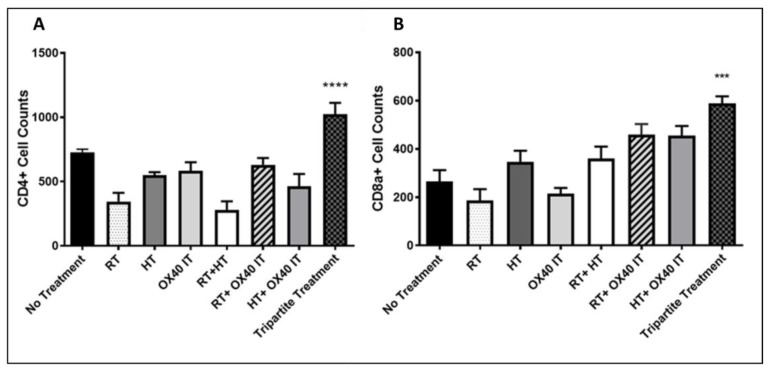
Flow cytometric analysis of tumor samples for tumor-targeting immune cells 10 days post treatment initiation. (**A**) Flow cytometry analysis and activation of CD4+ cell counts in control group (no treatment); individual and in different combinations of anti-OX40 IT, HT, and RT (treatment groups). (**B**) CD8a+ cell counts in control group (no treatment); individual and in different combinations of anti-OX40 IT, HT, and RT. All statistical analyses are presented compared to the control (significance shown by *); performed using 1-way ANOVA with multiple comparisons test. Statistical significance values demonstrated by respective symbols similar to *** *p* < 0.001 **** *p* < 0.0001. Data represent mean ± SEM.

**Figure 4:**
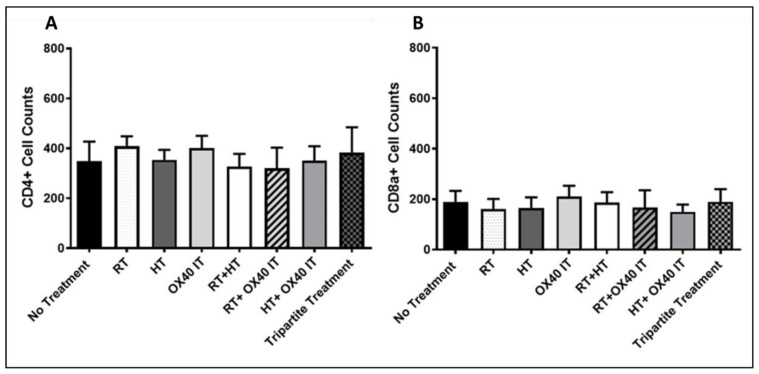
Flow cytometric analysis of tumor samples for tumor-targeting immune cells 45 days post treatment initiation. (**A**) Flow cytometry analysis and activation of CD4+ cell counts in control group (no treatment); individual and in different combinations of anti-OX40 IT, HT, and RT (treatment groups). (**B**) CD8a+ cell counts in control group (no treatment); individual and in combinations of anti-OX40 IT, HT, and RT. Data represent mean ± SEM.

The promising results on tumor growth delay obtained following tripartite treatment, prompted the investigation of the impact of immune response in the PC-TME. At 10 days post treatment initiation, flow cytometric analysis was performed to elucidate the early changes in the TME of all experimental animal groups. Flow cytometry measurement was performed using tumors digested lysate prepared from the euthanized animals at 10 days (early) and 45 days post-treatment which was the end of the study. After euthanasia, tumors obtained from the tripartite treatment groups showed an increased population of CD4+ and CD8a+ T-cells in the PC-TME as compared to the control group at 10 days post treatment initiation (Figure 3A,B). Concomitantly, there was a significantly higher influx of CD4+ (*p* < 0.0001) and CD8a+ (*p* < 0.001) T-cells in the TME of the tripartite treatment group as compared to any individual treatment group. Interestingly we also observed that the administration of RT significantly (*p* < 0.0001) reduced the resident population of CD4+ cells (Figure 3A,B) compared to no treatment group. Furthermore, combination therapy of RT + IT resulted in the significantly higher influx of CD4+ cells in the PC-TME (*p* < 0.01) compared to the control animals. Highest reduction in the CD4+ cell population was seen in the PC-TME of RT + HT treatment group animals (*p* < 0.0001) (Figure 3A). Interestingly, at 10 days post-treatment, HT + IT, RT + IT and tripartite treatment groups all showed increased level of CD8a-positive cells in the PC-TME compared to no treatment animals including monotherapies (Figure 3B). Tripartite treatment showed highly significant increase in CD8a-positive cells compared to no treatment (*p* < 0.05), and all individual treatment (*p* < 0.01) group. At 45 days post-treatment (end of the treatment), the population of CD4 and CD8a-positive cells decreased to the level of no treatment (control) and all mono- or bi-partite treatment combination groups (Figure 4A,B) and there was no significant differences were found in cell number between any of the groups.

### 2.4. Tripartite Treatment Does Not Alter the Myeloid Derived Suppressor Cells in the PC-TME

**Figure 5:**
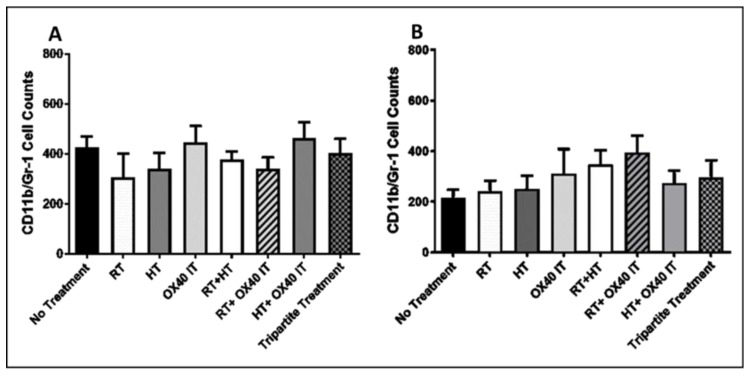
Flow cytometric analysis of tumor samples for CD11b/Gr-1-positive cell counts 10- and 45-days post treatment initiation. (**A**) Flow cytometry analysis and activation of CD11b/Gr-1-positive cell counts in control group (no treatment); individual and in different combinations of anti-OX40 IT, HT, and RT (treatment groups) at 10 days post-treatment. (**B**) CD11b/Gr-1-positive cell counts at 45 days post treatment in control group (no treatment); individual and in combinations of anti-OX40 IT, HT, and RT (treatment groups).

The expression of myeloid-derived suppressor cells (MDSCs) has an integral role in tumor immunosuppression, angiogenesis, drug resistance, and tumor metastasis. In our study, at 10 days post treatment initiation, no statistically significant changes in the CD11b+/Gr-1+MDSCs population were observed in any of the mono- or combination treatments (except HT + RT) groups compared to (no treatment) control group (Figure 5A). At the endpoint of 45 days post treatment initiation, the PC-TME showed no difference in CD11b/GR-1+ cells (Figure 5B) in any of the treatment groups as compared to control (no treatment animals TME).

## 3. Discussion

PC has an extensive desmoplastic reaction creating a stromal barrier that prevents the penetration of chemotherapy and immunotherapy agents. In addition, the stromal barrier prevents the infiltration of key immune cells, like cytotoxic CD8 T-cells. Hyperthermia presents a therapeutic modality with the potential to overcome this stromal barrier, allowing the penetration of systemic therapeutics (i.e., chemotherapy and immunotherapy) and infiltration of immune cells. In addition, hyperthermia functions as a radiosensitizer, enhancing the efficacy of radiation-induced cell killing. Therefore, hyperthermia at non-ablative temperatures (<43 °C) is not cytotoxic, and HT disposes of its functions by sensitizing TME to RT and IT and consequently augmenting tumor clearance.

Our pre-clinical syngeneic pancreatic cancer model demonstrates the ability of tumor-targeted hyperthermia to enhance immunotherapy combined with radiation therapy. Hyperthermia holds important clinical potential as it is already in use with patients, using radiofrequency, microwave, or ultrasound energy [27,28,29]. Further, in a prospective comparative cohort trial, Mulata et al. examined a cohort of 68 patients with locally-advanced pancreatic cancer that was treated with chemoradiotherapy (CRT) along with microwave-based hyperthermia (*N* = 40) or without hyperthermia (*N* = 28) [30]. Patients who underwent hyperthermia + CRT had an improved overall survival rate compared to CRT-alone patients, 15 months for CRT combined with hyperthermia vs. 11-months for CRT-alone. Importantly, hyperthermia was well tolerated with minimal toxicity. Further support for hyperthermia in the clinical setting for treatment of PC comes from a recent meta-analysis that demonstrated an improved median overall survival with hyperthermia + radiotherapy compared to radiotherapy alone, 11.7 vs. 5.6 months respectively [31].

Accumulating evidence suggests that immunotherapy alone exerts insufficient anti-tumor response to attain strong clinical responses in patients with PC. There are recent advances in checkpoint inhibitor-based immunotherapies such as anti-programmed death receptor (PD)-1 and anti-PD-ligand (L)-1 antibodies which significantly enhance anti-tumor T-cell activity with improved outcomes for patients with a variety of tumors including lung, melanoma, and head and neck cancers [32,33]. However, they have not shown any added benefit in PC patients possibly due to a highly immunosuppressive TME. Hence, novel treatment approaches are desperately needed to treat PC patients. In this present proof of principle investigation, we have shown that agonistic OX40 antibody in combination with HT and RT as tripartite treatment significantly inhibited PC-tumor growth until 45 days in a preclinical study. Consequently, combined tripartite treatment significantly enhanced animal survival, and enhanced anti-tumor response, which might be due to modulation and alteration in the immunosuppressive tumor microenvironment (TME) [10]. Nevertheless, it will be interesting to examine, if checkpoint inhibitors have some therapeutic benefits against immunosuppressive PC-TME, if combined with HT, RT, and IT as bi- or tripartite treatment modality.

PC stroma is very heterogeneous and comprised of ECM components including fibroblastic vascular cells, myofibroblasts, pancreatic stellate cells, immune cells, blood vessels, cytokines, and growth factors. Recent reports suggest that PC stroma serves as physical barrier to successful drug delivery to tumor site effectually [34,35], and it present one of the foremost challenges to the treatment of pancreatic cancer. Further, immunosuppressive TME is infiltrated by large numbers of potentially tumor-reactive effector T-cells and regulatory T-cells that show evidence of prior activation [36]. Most of these cells express the co-inhibitory molecule PD-1, however, blockade of its ligand, PD-L1 was unsuccessful in a small number of patients with locally-advanced-stage PC [37,38]. The lack of clinical efficacy of PD-L1 blockade in PC patients suggests that it may be necessary to address the immunosuppressive effects by employing immune co-stimulatory agents such as agonistic OX40 immunotherapy, combined with RT or hyperthermia (HT) [10,39]. OX40 (CD134) is expressed more in animals with PC and suppresses the activity of CD25+CD4+ T-reg cells, thereby activating effector T-cells. Anti-OX40 is a negative feedback inhibitor, and therefore, it is not subject to feedback inhibition like anti-CTLA4 or anti-PD-1 therapies [40,41]. Furthermore, HT also induces HSP70 synthesis, enhancing the effect of IL-2 in activating T-lymphocytes. In addition, HT increases recruitment of tumor-killing immune cells like natural killer (NK) cells, macrophages, and cytotoxic and helper T-cells, thereby increasing tumor antigenicity. Hence, in this study we used a syngeneic PC mouse model and tested the synergistic impact of tripartite treatment by combining fractionated RT, tumor targeted HT, and anti-OX40 immunotherapy (*p* < 0.001), with no tumor regrowth compared to non-treated animals as control group. In-flow cytometric data analysis of the tumor digest revealed significantly increases in the population of CD4+, and CD8a+ cells in the TME, compared to all individual treatment groups at 10 days post-treatment. It is envisaged that the tripartite treatment activates tumor antigen release and recruits immune cells which alleviate the immunosuppressive TME [10] in the early stage of tumor progression. Thus, it can be contemplated that HT in combination with RT and IT has an immune-stimulating potential that might result in strong anti-tumor immunity [10,42]. Furthermore, it is also envisaged that due to high stromal content surrounding the pancreatic tumor, therapeutically it is classified as cold tumor and could not respond to any single treatment modality. Conversely, the present findings have demonstrated as a proof of principle that tripartite treatment allow infiltration of CD4 and CD8a and other immune activators including effector T-cells and turns the pancreatic tumor to a hot tumor which is more responsive to tripartite treatment [10]. Further work is underway to characterize in more detail the mechanistic CD8 T-cell response, and synergy with hyperthermia, radiation therapy, and immunotherapy.

## 4. Methods

### 4.1. Development of Syngeneic Subcutaneous Tumor Model

Syngeneic PC cell line (Panc02) of mouse origin was injected subcutaneously into the right flank of 8–10-week-old male C57BL/6 mice (Charles River, Wilmington, MA, USA). Panc02 cells were obtained from Dr. Jeffrey Schlom, National Cancer Institute, National Institutes of Health, Bethesda, MD. The cells were grown and maintained in high glucose McCoy’s 5A Modified Media (Invitrogen), 10% FBS, 2 mM glutamine, 10,000 U/mL penicillin, 10,000 U/mL streptomycin, 1× non-essential amino acids, 10 mM HEPES, 1 mM sodium pyruvate, and 1 × 10^6^ cells per injection with Matrigel in 1:1 ratio for subcutaneous tumor (SC) inoculation into the mice. The University of Maryland Baltimore (UMB) Institutional Animal Care and Welfare Committee approved all animal handlings and procedures and in accordance with veterinarian recommendations for the proper care and use of laboratory animals. Tumor size/volume was assessed by electronic caliper with the following formula: V = [W^2^ × L]/2 where V is tumor volume, W is tumor width, L is tumor length (Figure S2). Once the tumor volume reached 50 mm^3^, 160 mice were randomized and assigned into two euthanasia points: (a) 10 days post randomization and (b) 45 days post randomization, and eight experimental groups (*N* = 10): (1) no treatment, (2) RT, (3) HT, (4) anti-OX40 immunotherapy (Bioxcell, West Lebanon, NH, USA, Clone: OX-86, catalog # BE0031), (5) RT with HT, (6) RT with anti-OX40 immunotherapy, (7) HT with anti-OX40 Immunotherapy, and (8) tripartite treatment (RT with HT and anti-OX40 immunotherapy). Tumor size and body weight of the mice were measured every third day till 45 days post treatment initiation (Figure 1C). Animals were euthanized at their study end points of 10 and 45 days after any treatment initiation accordingly to by the University of Maryland Institutional Animal Care and Use Committee (IACUC protocol # 0816010) and in accordance with veterinarian recommendations for the proper care and use of laboratory animals.

### 4.2. Tumor Targeted Hyperthermia (HT)

A water bath with a circulating water heater was used to maintain water temperature at 42.5 °C. The water temperature was set to achieve a temperature of 42.5 °C in the subcutaneous tumor core. The target intra-tumoral temperature was achieved and optimized by measuring the tumor core temperature with a type K thermocouple temperature. Once the mice were anesthetized with isoflurane, they were placed on a 3D-printed customized plastic platform over the water bath and restrained to prevent movement for an accurate and robust administration of heat (Figure S1). The right hind limb with the tumor was lowered into the water bath completely immersing the tumor in the heated water maintained at 42.5 °C for a period of 30 min for each installment of the HT treatment (Figure 1C, Figure S1). As a pilot study, we performed immunohistochemistry of heat shock protein (HSP-70) in control and heated tumors and found HSP expression uniformly in heated tumors (Figure S4). 

### 4.3. Tumor Targeted Radiation (RT)

The Small Animal Radiation Research Platform (SARRP, Xstrahl Ltd., Camberley, UK) was used for cone beam computer tomography (CBCT) imaging and RT. For CBCT, anesthetized mice were placed stomach down onto the animal platform of the SARRP and secured in place. The nose cones of the isoflurane system were placed over the nose and mouth of the mice. The hind limb region with the tumor was imaged using the SARRP′s onboard CT scanner. The SARRP software reconstructed a 3D image of the region, and the tumor was identified. The center of the radiation field was placed on the center of the identified subcutaneous tumor. RT was delivered in four axial beams, collimated to a 1-cm-diamter circular field at the target isocenter. The delivered dose to the SC tumor region was 8 Gy in two fractions with a day interval. The maximal energy used to generate the X-rays was 225 kVp and 13 mA with a dose rate of 0.3 Gy/min. Absolute dosimetry was determined following the American Association of Physicists in Medicine (AAPM) TG-61 protocol for kilovoltage X-ray beam dosimetry [43].

### 4.4. Immunotherapeutic Treatment (IT)

Agonistic mouse OX40 monoclonal antibody (BioXcell, West Lebanon, NH, USA) was injected intraperitoneally (IP) at a 200 ug/mouse dose [44,45]. The first injection was administered after the completion of the RT dosage and thereafter two additional doses were administered every 5 days.

### 4.5. Flow Cytometry

Tumors were collected at euthanasia at two different points (10 days and 45 days post-treatment). Tissues were then collected, and single cell suspensions were obtained for flow cytometry analysis [31]. Briefly, tumors were processed through a cell strainer and washed in PBS 2% FBS (Gemini) 1% PenStrep 1% NEAA (Gibco, Life Technologies, Gaithersburg, MD, USA). Cells were stained at the supplier recommended concentrations of the antibody for 30 min at 4 °C in 100 μL FACS buffer. All antibodies were from BioLegend (San Diego, CA, USA). Two panels of mouse monoclonal antibodies were used; Panel 1: CD4 (clone GK 1.5, product # 100421), CD8a (clone 53-6.7, product # 100705) and Panel 2: Ly6G/Ly6C (Gr-1) (clone RB6-8C5, product # 108420) and CD11b (clone M1/70, product # 101205). All flow cytometry was performed at the University of Maryland Greenebaum Cancer Center Flow Cytometry Shared Services on the BD LSR II and high throughput sampler (HTS). Flow cytometry acquisition [45], was performed using a LSRII instrument (BD Biosciences) and data were analyzed using FlowJo software (Version 10.6, Tree Star Inc., Ashland, OR, USA).

### 4.6. Statistical Analysis

Statistics were analyzed by one-way ANOVA, two-way ANOVA, and correlation analysis by Prism (Graphpad) or IBM SPSS. The tumor growth delay was evaluated for statistical significance by one-way ANOVA coupled with a multiple comparison Tukey’s analysis of the column means, which demonstrated a *p*-value of less than 0.05. Animal survival was plotted on a Kaplan–Meir survival plot to demonstrate the survivability of each individual treatment.

## 5. Conclusions

Consequently, in this present “proof of concept” study, tripartite treatment conspicuously mitigated the immunosuppressive TME to an immune responsive one. In addition, HT is also known to delay DNA repair in cancer cells, thereby enhancing RT-induced tumor killing of cancer cells. Further, RT helps in simulating and release of tumor antigen production which might be responsible for coherent immune assault which may help in controlling PC (Figure 1A,B). We also conclude that RT could turn an immunotherapeutically-nonresponsive tumor into a rather responsive tumor and enhance efficacy of anti-OX40 IT [10]. To our knowledge, this study provides the first proof of principle that combining HT, RT, and anti-OX40 IT propagates a robust anti-tumor response in mice. Further investigations are needed to delineate the role of (a) cellular components (regulatory cells, natural killer cells, etc.) that include costimulatory versus coinhibitory molecules, (b) molecular components (CD28, CD86, CD80, etc.), and (c) cellular mechanisms involved in anti-tumor response. Furthermore, investigations are also required to test it in another pancreatic tumor model, optimize doses, and sequence of tripartite treatment to treat both locally-advanced and metastatic PC to provide the rationale for future clinical trials.

## Supplementary Materials

The following are available online at https://www.mdpi.com/2072-6694/12/4/1015/s1: Figure S1: Three-dimensional model of hyperthermia administration platform and isoflurane nose cone setup, Figure S2: Tripartite treatment showed significant tumor size reduction at 45 days post-treatment initiation compared to non-treated control animals, Figure S3: All treatment group showed significant tumor weight reduction (*p* < 0.05) at 45 days compared to non-treated control animals, and Figure S4: Immunohistochemistry of heat shock protein-70 expression in mouse subcutaneous PC tumor.
