# Supplementary material for: A Combination of Radiotherapy, Hyperthermia, and Immunotherapy Inhibits Pancreatic Tumor Growth and Prolongs the Survival of Mice
Source: Cancers (Basel). 2020 Apr 21;12(4):1015. doi: 10.3390/cancers12041015 (PMC7226594; doi:10.3390/cancers12041015)
Supplement: Supplementary file 1 [file cancers-12-01015-s001.pdf]

cancers

MDPI

Article

A Combination of Radiotherapy, Hyperthermia, and Immunotherapy Inhibits Pancreatic Tumor Growth and Prolongs the Survival of Mice

Javed Mahmood, Allen A. Alexander, Santanu Samanta, Shriya Kamlapurkar, Prerna Singh, Ali Saeed, France Carrier, Xuefang Cao, Hem D Shukla and Zeljko Vujaskovic

Supplementary Materials

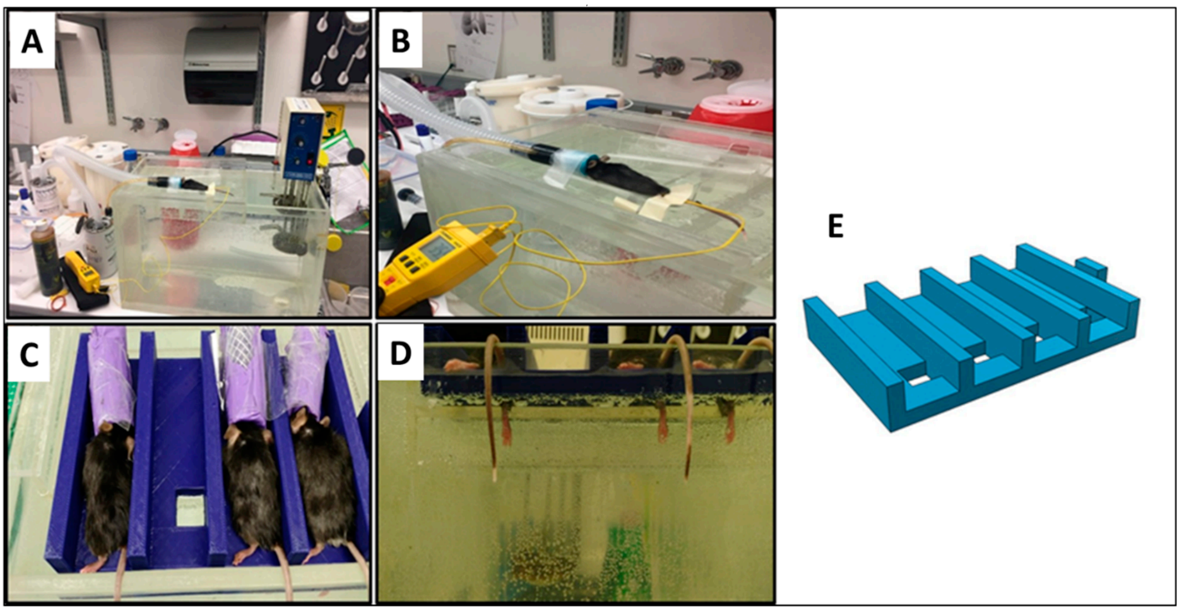

Figure S1. Three-Dimensional model of hyperthermia administration platform and isoflurane nose cone setup. (**A**). Water bath setup with heating coil circulator and isoflurane vaporizer (**B**). Temperature thermocouple measuring water temperature (**C**). Mice under anesthesia receiving hyperthermia while lying on the 3D printed hyperthermia administration platform (**D**). Front view of mice legs immersed in the water bath during hyperthermia administration. (**E**). Different angles of custom-made 3D model platforms to administer hyperthermia to the mice tumor precisely.

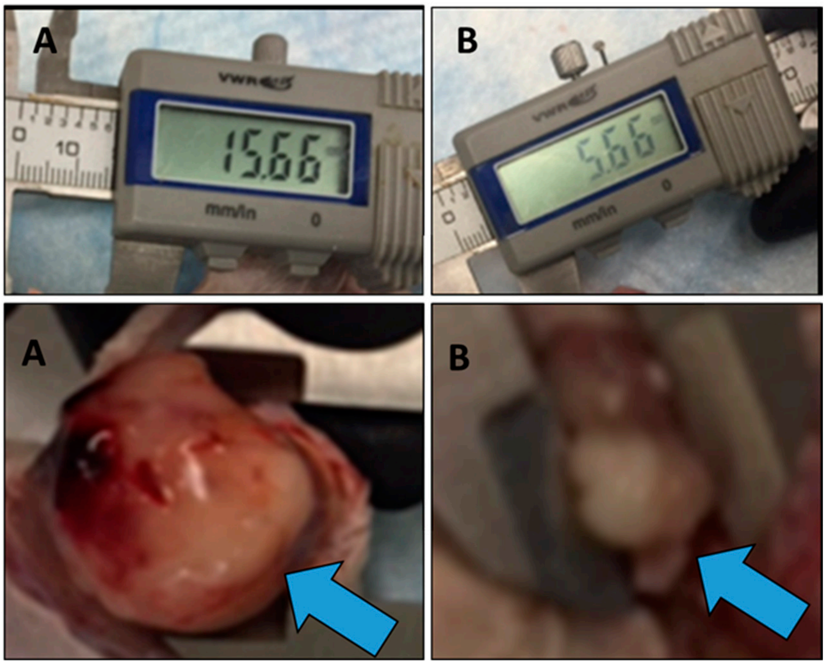

Figure S2. Tripartite treatment showed significant tumor size reduction at 45 days post-treatment initiation compared to non-treated control animals. (**A**). Non-treated Control tumor after euthanasia; tumor length 1.56 cm. (B). Tripartite treated tumor after euthanasia; tumor length 5.6 cm measured using slide caliper. Arrow showing the actual tumor in the mice.

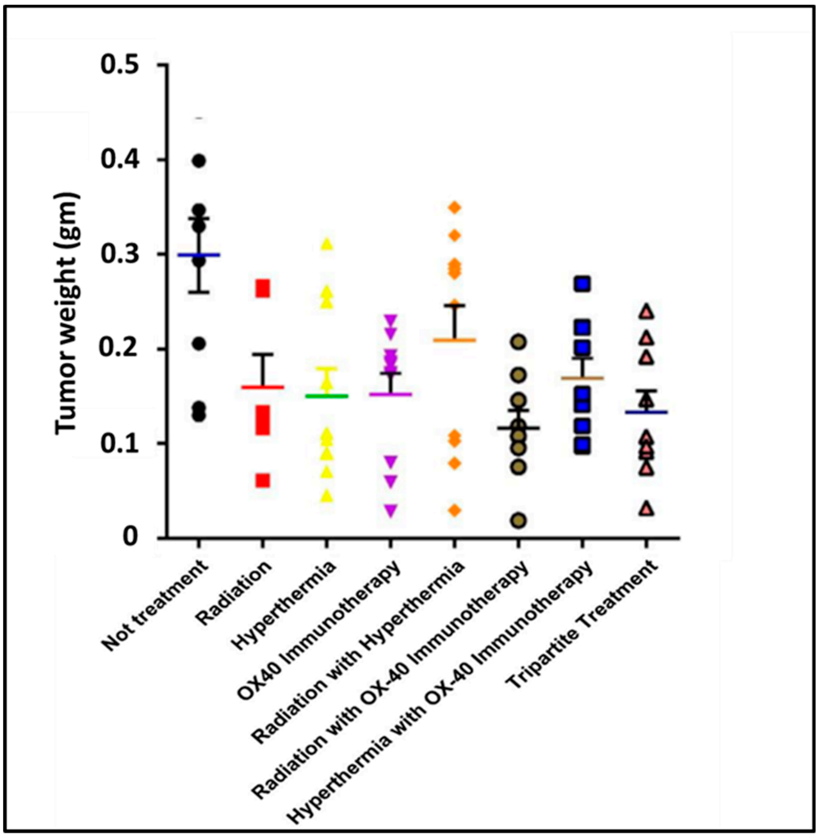

Figure S3. All treatment group showed significant tumor weight reduction ($p < 0.05$) at 45 days compared to non-treated control animals.

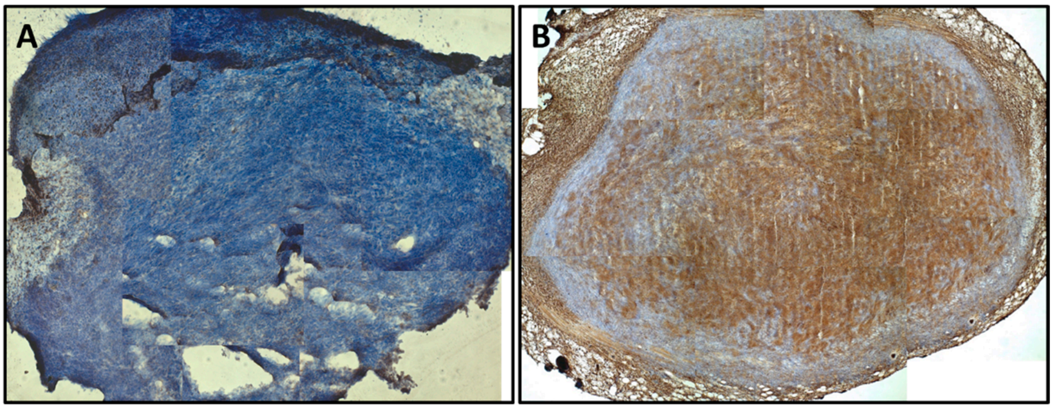

Figure S4. Immunohistochemistry of Heat shock protein-70 expression in mouse subcutaneous PC tumor (**A**). Un-treated Control (**B**). Tumor targeted hyperthermia.

© 2020 by the authors. Licensee MDPI, Basel, Switzerland. This article is an open access article distributed under the terms and conditions of the Creative Commons Attribution (CC BY) license (http://creativecommons.org/licenses/by/4.0/).
